# Fluorescent Chemosensors for Toxic Organophosphorus Pesticides: A Review

**DOI:** 10.3390/s100707018

**Published:** 2010-07-21

**Authors:** Sherine O. Obare, Chandrima De, Wen Guo, Tajay L. Haywood, Tova A. Samuels, Clara P. Adams, Noah O. Masika, Desmond H. Murray, Ginger A. Anderson, Keith Campbell, Kenneth Fletcher

**Affiliations:** 1 Department of Chemistry, Western Michigan University, Kalamazoo, MI 49008, USA; E-Mails: chandrimade@gmail.com (C.D.); wen.guo@wmich.edu (W.G.); tajay.haywood@wmich.edu (T.L.H.); tova.a.samuels@wmich.edu (T.A.S.); clara.p.adams@wmich.edu (C.P.A.); noah.o.masika@wmich.edu (N.O.M.); gingeraa@umich.edu (G.A.A.); 2 Department of Chemistry and Biochemistry, Andrews University, Berrien Springs, MI 49104, USA; E-Mails: murrayd@andrews.edu (D.H.M.); ebullientc@netscape.net (K.C.); fletcha@msn.com (K.F.); 3 Building Excellence in Science and Technology, Berrien Springs, MI 49103, USA

**Keywords:** organophosphorus compounds, fluorescent chemosensors, pollutants, dual modes of signal transduction

## Abstract

Many organophosphorus (OP) based compounds are highly toxic and powerful inhibitors of cholinesterases that generate serious environmental and human health concerns. Organothiophosphates with a thiophosphoryl (P=S) functional group constitute a broad class of these widely used pesticides. They are related to the more reactive phosphoryl (P=O) organophosphates, which include very lethal nerve agents and chemical warfare agents, such as, VX, Soman and Sarin. Unfortunately, widespread and frequent commercial use of OP-based compounds in agricultural lands has resulted in their presence as residues in crops, livestock, and poultry products and also led to their migration into aquifers. Thus, the design of new sensors with improved analyte selectivity and sensitivity is of paramount importance in this area. Herein, we review recent advances in the development of fluorescent chemosensors for toxic OP pesticides and related compounds. We also discuss challenges and progress towards the design of future chemosensors with dual modes for signal transduction.

## 1. Introduction

Environmental pollution by organic chemicals continues to be one of the world’s leading challenges to sustainable development. Modern developed and developing countries utilize millions [1] of synthetic organic compounds in their civilian, commercial, and defense sectors for an ever-expanding diversity of uses. Common applications include plastics, lubricants, refrigerants, fuels, solvents, preservatives, surfactants, dispersants and pesticides. As a result of widespread global usage coupled with improper handling practices, many of these organic compounds enter the environment and cause air, water, and soil pollution. For example, pesticides and herbicides are applied directly to plants and soils, while accidental releases originate from spills, leaking pipes, underground storage tanks, waste dumps, and waste repositories. Many pesticides are sprayed in large amounts with only 1% reaching the intended target. Some of these contaminants have long half-lives and thus persist to varying degrees in the environment. They migrate through large regions of soil until they reach water resources, where they may present an ecological or human-health threat [2]. Organisms, vegetation, animals and humans are affected by various chemicals through absorption, inhalation or ingestion. These contaminants pose serious to fatal health hazards, such as asthma, birth defects and deaths. Therefore, environmental monitoring is required to protect the public and the environment from possible organic toxins released into the air, soil, and water.

The United States Environmental Protection Agency (U.S. EPA) has imposed strict regulations on the concentrations of many environmental contaminants in air and water [3]. However, current monitoring methods for most organic contaminants are costly and time-intensive, and limitations in sampling and analytical techniques exist [4]. Thus, there is a great demand for development of quick, simple and reliable methods for the detection of organic-based agricultural pesticides. In this review article we describe fluorescent chemosensors designed to detect OP pesticides and related compounds. We also discuss the importance of developing multimodal chemosensors (*i.e.*, with more than one mode of signal transduction) for real-time detection of OP compounds.

### 1.1. Structure of Organophosphorus Compounds

**Figure 1.**
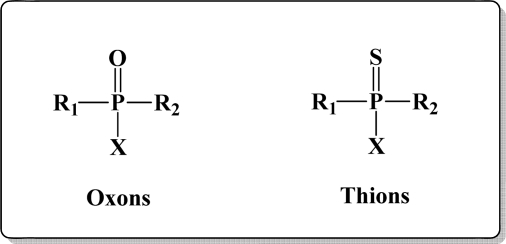
General chemical structure of oxon and thion OP compounds.

OP pesticides are synthetic esters, amides, or thiol derivatives of phosphoric, phosphonic, phosphorothioic, or phosphonothioic acids. There are over 100 OP compounds currently in the market, representing a variety of chemical, physical, and biological properties. Table 1 lists the names of the most commonly used OP pesticides, which is in part the subject of this review [5–7]. As the name indicates, all OP pesticides have a central phosphorus atom, with either double bonded oxygen (P=O), or a double bonded sulfur atom (P=S). AP=O pesticide is called an oxon pesticide, and the P=S is termed as a thion pesticide as shown in Figure 1.

Structurally, both oxons and thions show variety in the single-bonded R_1_, R_2_ and X groups attached to the central pentavalent phosphorus atom. However, R_1_ and R_2_ generally tend to be alkoxy, aryloxy and thioalkoxy groups, while X is a labile leaving group. TEPP, the most potent oxon pesticide in Table 1, has a unique pyrophosphate structural motif, a biochemically important high energy phosphate bond. The most potent thion pesticide, parathion, has a *p*-nitrophenoxy (X) substituent, which is a very good leaving group in nucleophilic substitution reactions involving the central phosphorus atom.

### 1.2. OP Compounds and Their Toxicity

Pesticides are described as chemicals that kill or slow down the growth of undesirable organisms. Pesticides include herbicides, insecticides, fungicides, and nematocides. Nowadays, it is believed that application of synthetic pesticides is one of the most effective methods for controlling insects that affect crop growth [8]. OP pesticides constitute the most widely used insecticides available today. This class of compounds has achieved enormous commercial success as a key component in the arsenal of agrichemicals, and is currently an integral element of modern agriculture across the globe. According to the EPA, about 70% of the insecticides in current use in the US are OP pesticides [3]. They were developed to replace organohalide pesticides in the late 1950’s because OP pesticides are relatively easier to degrade via microbial or environmental processes. Unlike organohalide pesticides, the OP pesticides do not bioaccumulate due to their rapid breakdown in the environment and they are thus preferred over organohalides for insecticide and/or pesticide use.

Although OP compounds are considered safer than organohalides, they are still highly neurotoxic to humans and in some cases their degradation products have the potential to be more toxic with chronic exposure. OP compounds are efficiently absorbed by inhalation, ingestion, and skin penetration. They are strong inhibitors of cholinesterase enzymes that function as neurotransmitters, including acetylcholinesterase, butylcholinesterase, and pseudocholinesterase. These enzymes are inhibited by binding to the OP compound. Upon binding, the OP compound undergoes hydrolysis leading to a stable phosphorylated and a largely unreacted enzyme. This inhibition results in the accumulation of acetylcholine at the neuron/neuron and neuron/muscle junctions or synapses.

Each year OPs poison thousands of humans across the world. In fact, in 1994, an estimated 74,000 children were involved in common household pesticide related poisoning or exposures in the United States [3]. In a more recent study, it was found that children exposed to OP pesticides were more likely to be diagnosed with attention deficit hyperactivity disorder (ADHD) [9]. Exposure has been attributed to frequent use of OPs in agricultural lands and their presence as residues in fruits, vegetables, livestock, poultry products and municipal aquifers [10]. For example, typical pesticide concentrations that flow into aqueous waste range from 10,000 to 1 ppm [4,11].

**Figure 2.**
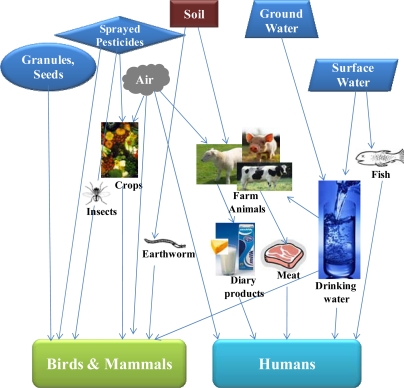
Schematic representation of the possible routes of environmental exposure of OP pesticides to humans and wildlife. Adopted from Reference [12].

Pesticides are influenced by a number of biological, chemical and physical processes once they enter the environment. Figure 2 shows the possible routes of environmental exposure of organo-phosphorus pesticides to humans and wildlife [12]. While many OP pesticides can degrade via microbial or environmental processes, some of the pesticides are consumed by organisms, or they could leach into ground water. Once a pesticide enters ground water it can remain there for considerable periods of time. In ground water, there is little sunlight exposure, which slows down the degradation of OP pesticides and increases their potential risks to the environment and human health.

The first indication of insecticidal activity among OP compounds was found in 1930, however, the first compound of this type, hexaethyl tetraphosphate (HETP) [an impure mixture containing tetraethylpyrophosphate (TEPP) as the active ingredient] was not used as an agricultural insecticide until 1942 [13]. Since the first introduction of HETP, the number of OP pesticides has risen to hundreds, and the common ones are shown in Table 1, along with their toxicity information. As indicated by their LD_50_ values in Table 1, there are some significant bioactivity differences between oxons and thions. Generally, compared to thion pesticides, the oxons are more potent with lower LD_50_’s. One of the most potent oxon pesticides is TEPP with an oral LD_50_ of 0.5 mg/kg, while one of the most active thion pesticides is parathion with an oral LD_50_ of 1 mg/kg. In general, Table 1 indicates that the oral toxicity for an individual OP is much greater than their dermal toxicity.

## 2. Advances in Detection of OP compounds

Significant advances toward the development of detection methods for OP compounds have been reported in the literature [14–20]. Analysis of OPs in environmental and biological samples is routinely conducted using various analytical techniques, including nuclear magnetic resonance (NMR) spectroscopy [21], gas, liquid or thin layer chromatography, and mass spectrometry [20]. A variety of approaches have been investigated for sensors, including enzymatic assays [18], molecular imprinting coupled with luminescence (using lanthanides) [14–17], colorimetric methods [22–24], surface acoustic waves [25,26], fluorescent organic molecules [27–29], and interferometry [19]. The most common ways for detecting OP pesticides are chromatographic methods coupled with different detectors and different types of spectroscopy, immunoassays, and enzyme biosensors based on inhibition of cholinesterase activity [30–32].

An alternative to classical methods for detection of OP pesticides is the design of optical sensors, *i.e.,* colorimetric or fluorimetric chemosensors or reagents. One of the most convenient and simple means of chemical detection is the generation of an optical signal, for example, changes in absorption or emission bands of the chemosensor in the presence of the target analyte. Optical outputs have been used extensively in recent years for the development of chemosensors for ion or neutral molecule recognition and sensing based on supramolecular concepts [33]. Unfortunately, although the utility of optical detection is becoming increasingly appreciated in terms of both qualitative and quantitative analysis, the number of optical sensors currently available for OP compound detection is quite limited.

Fluorescence-based sensors, both biosensors and chemosensors, offer significant advantages over other conventional methods for detection of OP compounds. The principal advantages of fluorescence are its high single-molecule sensitivity and in some most cases almost instantaneous response. Fluorescence methods are capable of measuring concentrations of analytes 10^6^ times smaller than absorbance techniques [33]. Thus, fluorescence techniques have been widely used in molecular biology and analytical chemistry but not extensively in the detection of OP pesticides.

### 2.1. Fluorescence-based Biosensors for OP Compounds

To date, a number of sensitive biosensors based on acetylcholinesterase (AChE) or butyryl cholinesterase (BChE) inhibition have been developed and used for OP compound detection [18,30,34–41]. In general, enzyme-based sensors for the detection of OP compounds can be broadly categorized into two major classes based on the enzyme employed-(1) AChE or (2) organophosphorus hydrolase (OPH).

**Figure 3.**
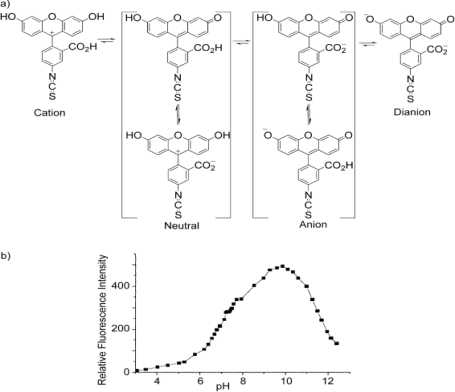
(a) Structure of fluorescein isothiocyanate (FITC) at different pH, (b) its relative fluorescence intensity at selected pH values. Reproduced with permission from reference [44], published by Elsevier, 2004.

Hydrolysis of acetylcholine by AChE produces one proton per substrate molecule resulting in an increase in the acidity of the solution. This forms the basis for AChE-based sensors. Rogers *et al*. [41] used a pH-sensitive fluorescent dye, consisting of AChE linked to the pH-sensitive compound fluorescein isothiocyanate (FITC). The enzyme-dye adduct was immobilized on a quartz fiber which was attached to a fluorescence spectrophotometer. In the absence of an OP compound, the labeled AChE was able to hydrolyze acetylcholine leading to a decrease in pH which resulted in the reduction of the FITC fluorescence intensity due to interruption of the fluorophore’s conjugation upon protonation (Figure 3). However, in the presence of diisopropylfluorophosphate (DFP) and subsequently acetylcholine, it was observed that 90% of the enzyme activity was lost, which was quantified by a less pronounced reduction of the fluorescence intensity. This biosensor was found to be very sensitive (capable of detecting nanomolar (nM) concentrations of paraoxon when exposed to the solution containing the analyte for ten minutes), and it demonstrated some selectivity toward different OP compounds.

**Figure 4.**
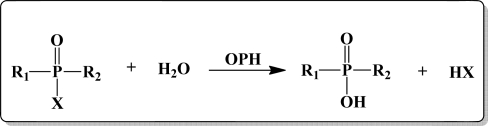
Mechanism for the hydrolysis of OP compounds by OPH.

The second family of biosensors utilizes OPH as the enzymatic sensor for the detection of OP compounds. The mode of action of OPH is different from AChE; it catalytically hydrolyses the OP compound, as illustrated in Figure 4, instead of covalently binding to it. Thus, instead of measuring the enzyme inhibition, detection methods involving OPH allow for a more direct measurement of OP compounds. Nowadays, OPH is widely used as a biosensor because of its ability to hydrolyze a wide range of compounds containing P-O, P-F, P-S, or P-CN bonds [40,42]. Hydrolysis of the OP compounds led to the stoichiometric production of two protons which can be monitored and directly correlated to the amount of OP substrate [43]. For instance, Cao *et al.* [38] labeled OPH with FITC and deposited the resulting material onto silanized quartz slides in the form of Langmuir-Blodgett films thus creating organized monolayers of the enzyme-based sensors. It was demonstrated that this OPH based enzyme sensor showed enhanced sensitivity and could detect the analyte at nM concentrations.

A number of biosensors have been developed based on fluorescence polarization immunoassays (FPIA) [45–48]. One example reported by Kolosova *et al*. showed the use of a monoclonal antibody for the detection of parathion-methyl using FPIA [45]. The sensing unit comprised a parathion-methyl derivative linked to fluorescein. Binding to parathion methyl or other closely related compounds was confirmed by measuring the intensity of emitted polarized light which indicated antibody binding. Despite the susceptibility of interference with different components existing in some matrices and the wide determinative range, the FPIA method is highly specific and reproducible and without complicated cleanup the method meets the performance criteria for detecting parathion-methyl.

In summary, enzyme based sensors are both very sensitive and selective in their approach to detect OP compounds. Furthermore, OPH based enzyme sensors offer distinct advantages over AChE-based systems. While these approaches towards OP detection have been significant, the inhibition-based biosensors suffer from three drawbacks: (1) the enzymes easily lose activity in the event of environmental or handling factors, therefore these enzymes may provide false positive signals, (2) the sensors require baseline testing prior to sample application and lengthy incubation times to allow enzyme-analyte interaction, and (3) due to the irreversible nature of cholinesterase enzyme inhibition, inhibition-based sensors cannot be reused without regeneration of enzyme activity. In addition, the lifetime of these sensors is limited by enzyme degradation.

### 2.2. Fluorescence-based Chemosensor Detection Methods

**Figure 5.**
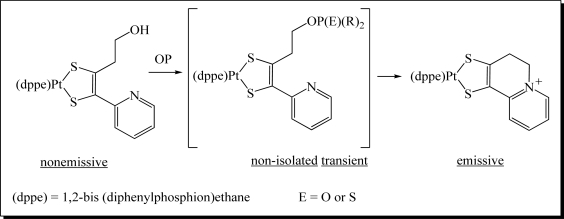
Mechanism of the chemically reactive sensor developed by Van Houten *et al*. Reproduced with permission from Reference [29], published by American Chemical Society, 1998.

Recently, a number of innovative methods for the detection of OP compounds based on optical chemosensors have been reported in the literature. The first fluorescent chemosensor for detection of OP compounds was reported by Van Houten *et al*. [29] where a series of non-emissive platinum 1,2-enedithiolate complexes with an appended primary alcohol were synthesized. Upon addition of electrophilic OP analyte to this compound and an activation agent (triazole) in dichloromethane, the alcohol was converted to a phosphate ester, which reacts intramolecularly to form a fluorescent cyclic product (Figure 5). The method was found to be very effective in the detection of a variety of nerve agents. The analysis was conducted with care of avoiding oxygen since the presence of oxygen quenched the fluorescence.

Zhang and Swager [49] developed a series of thienylpyridyl and phenylpyridyl systems which undergo intramolecular cyclization reactions upon exposure to OP compounds. Binding resulted in spectral bathochromic shifts in the absorption and fluorescence of these chemosensors. Notable fluorescence color changes were observed using a UV lamp under ambient atmosphere. These sensors were found to be both sensitive and selective to OP compounds showing a complete response to 10 ppm (diisopropylfluorophosphate) DFP vapor within five minutes.

**Figure 7.**
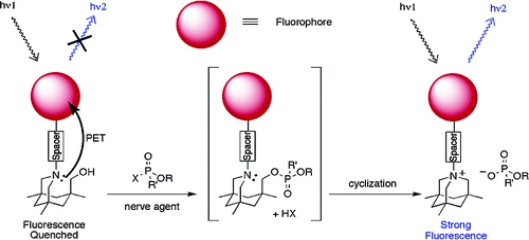
The chemically reactive sensor reported by Rebek’s group. Taken with Reproduced with permission from Reference [50], published by American Chemical Society, 2006.

Rebek’s group [50] carried out similar work to develop sensors for OP compounds where a series of pyrene based compounds were examined as possible fluorescent receptors (Figure 7). The design of the chemosensors were similar to that shown by Van Houten *et al*. and Zhang and Swager, however the binding reaction mechanism was based on the suppression of a photoinduced electron transfer (PET) process to trigger a fluorescence signal. Saturated aliphatic chains ranging from one to four methylene units were employed to determine how the spacer linking the fluorophore and the amine affected the efficiency of the sensors. Pyrene was used as the fluorophore since it can accept electrons from tertiary amines in PET processes. Upon binding to diethylchlorophosphate (DCP), the sensors resulted in a significant increase in fluorescence intensity and the changes were visible to the naked eye when the samples were viewed which could be observed visually using a handheld UV lamp. The sensor displayed an instantaneous (within 5 seconds) fluorescence upon exposure to as little as 10 ppm DCP vapor.

**Figure 8.**
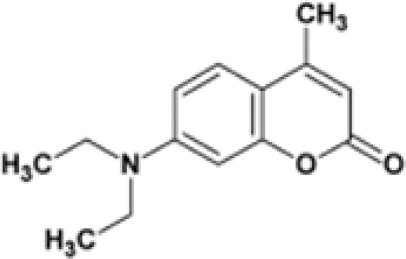
Coumarin 1, fluorescent compound and inhibitor reported by Simonian’s group. Reproduced with permission from reference [51], published by Elsevier, 2007.

In 2007, Simonian’s group [51] reported a fluorescence based sensor for OP pesticides based on Coumarin 1 which is shown in Figure 8. Coumarin 1 in the presence of *p*-nitrophenol-substituted OP compounds leads to fluorescence quenching due to fluorescence resonance energy transfer (FRET). The sensor is very effective in the detection of nitrophenyl substituted pesticides like methyl parathion and fenitrothion.

**Figure 9.**
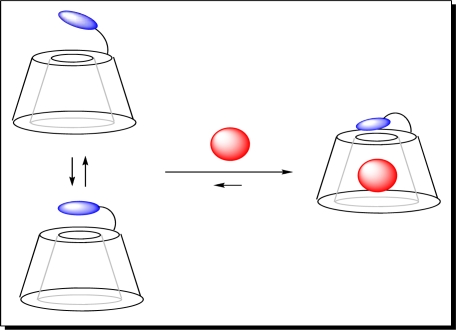
Inclusion phenomena of a guest in CDs molecules. Reproduced with permission from Reference [52], published by Bentham Science, 2009.

Delattre and co-workers [52] reported a cyclodextrin (CD) based fluorescent sensor for the detection of pesticides in water. d-Glucopyranose units in CDs form truncated cone-shaped molecules with a hydrophobic cavity, which can induce the inclusion phenomena of a guest, as shown in Figure 9. The dipole of the macromolecular system varies with the entry of a guest molecule. A modified β-cyclodextrin, pyridinoindolizin-β-cyclodextrin, was used to detect pesticides and herbicides, linadane, parathion, malathion, imidacloprid, atrazine, and simazine, through an inclusion complex between the pesticide or herbicide and the hydrophobic cavity of the macrocycle. This interaction leads to fluorescence quenching of the fluorophore. An advantage of this fluorescence sensor is the ability to quantify concentration data via fluorescence intensity concentration-dependence.

**Figure 10.**
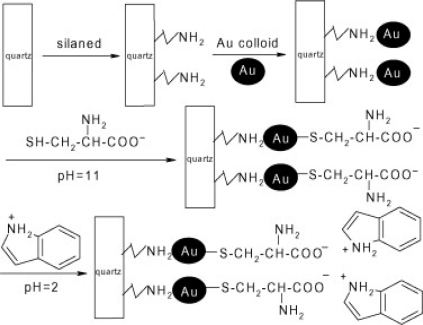
Schematic representation of the formation of the indole-based SAM sensor. Reproduced with permission from Reference [53], published by Elsevier, 2008.

A self-assembled multilayer (SAM) consisting of amino-silanized quartz functionalized with gold nanoparticles and coated with indole via a l-cysteine linker was fabricated as shown in Figure 10 [53]. When the SAM sensor was exposed to the pesticide, the indole group of the sensor on the modified film was oxidized to a fluorescent indoxyl group. The oxidation process depended on the pesticide concentration and was reflected by changes in intensity. The sensor was capable of detecting methylparathion and monocrotophos in the ppm and ppb range, respectively. An advantage of the indole-based SAM sensor is that it could detect OP pesticides in ionic and other environmental species, but it was subject to interference at 20 equivalents of Fe^3+^ ions.

## 3. Sensors with Multiple Modes of Signal Transduction

There is a growing awareness and trend toward the development of multimodal systems reminiscent of living organisms that utilize multiple senses to intelligently respond to multiple stimuli in real-world environments. A major advantage of mutimodal sensors is the minimization of false positives. With this in mind, we have recently developed and reported new chemosensors with multimodal sensing capabilities for analytes such as saccharides [54] and toxic OP compounds [55].

**Figure 11.**
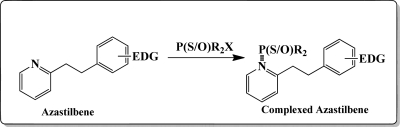
Schematic representation of uncomplexed (*left*) and complexed (*right*) azastilbene [55]. (EDG = electron donating group).

Our design strategy, shown in Figure 11, utilizes and couples the electrophilic reactivity of the pentavalent phosphorus atom of the phosphoryl and thiophosphoryl groups of toxic OPs to a nucleophilic fluorophore capable of recognizing and reporting sensor—analyte interactions. Signal transduction was anticipated to occur best via the π-electronic system of a donor acceptor azastilbene upon complexation of the electrophilic phosphorus to the nucleophilic binding site of our optical chemosensor.

**Figure 12.**
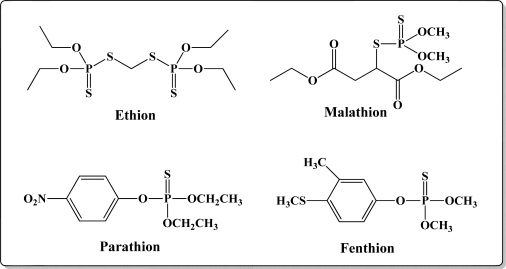
Chemical structures of ethion, malathion, parathion, and fenthion pesticides.

**Scheme 1.**
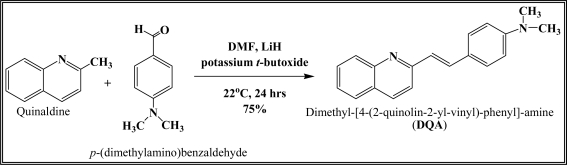
Schematic representation for the synthesis of DQA.

Figure 12 shows the structure of the OP pesticides used in our study to evaluate the effectiveness of donor acceptor azastilbenes as sensors. We have shown that the azastilbene, dimethyl-[4-(2-quinolin-2-yl-vinyl)-phenyl]-amine (DQA), recognizes, reacts with and responds to the pesticides: ethion, malathion, parathion, and fenthion. DQA binding to either of the above mentioned pesticides resulted in changes of the UV-visible, fluorescence and cyclic voltammogram of DQA [55] indicating the selective binding. To synthesize donor-acceptor azastilbene chemosensors we relied upon the single step approach involving base-promoted condensation of donor-substituted aromatic aldehydes with methyl-substituted azaaromatics [56–58]. DQA was made in 75% yield by room temperature condensation of a 1:1 quinaldine and 4-dimethylaminobenzaldehyde mixture (Scheme 1). Excess potassium *tert*-butoxide base was used along with lithium hydride which ensured a dry non-protic reaction medium.

**Figure 13.**
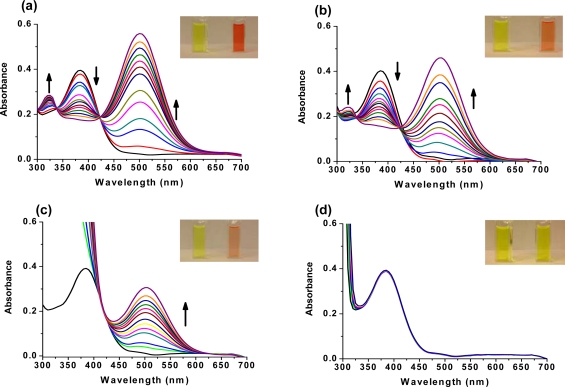
Changes in UV-visible absorbance of DQA upon binding to OP pesticides: (a) titration with ethion; (b) titration with malathion; (c) titration with parathion; and (d) titration with fenthion. In each case the direction of the arrow indicates concentration of 0, 2, 4, 6, 8, 10, 12, 14, 16, 18, 20, 22, 24 μM.

The interaction of DQA with ethion, malathion, parathion and fenthion was studied by different methods, including (1) UV-visible absorbance spectroscopy, (2) fluorescence spectroscopy, and (3) cyclic voltammetry. A solution of DQA in acetonitrile absorbs at 385 nm which corresponds to an intramolecular charge transition from the dimethylamine nitrogen to the quinaldine nitrogen [59]. DQA was titrated with each pesticide and changes in the UV-visible absorbance spectrum of DQA were measured. As shown in Figure 13a, increase in ethion concentration to a solution of DQA in acetonitrile resulted in the decrease in the UV-visible absorbance intensity at 385 nm, and was accompanied by formation of two new peaks at 325 nm and at 500 nm. Similar behavior was observed in the case of malathion, except two new peaks arise at 330 nm and 505 nm, as shown in Figure 13b. In both cases two isosbestic points were observed at 340 nm and 425 nm for ethion, and at 335 nm and 430 nm for malathion. Furthermore, we observed that titration of parathion to a solution of DQA in acetonitrile did not result in the quenching of the 325 nm peak, however, a new peak at 505 nm formed and increased in intensity with an increase in parathion concentration as shown in Figure 13c. On the other hand, addition of fenthion to the DQA solution did not show any notable changes in the original absorbance of DQA as shown in Figure 13d. Changes in the UV-visible absorbance spectrum show that DQA is efficient in distinguishing between the four OP pesticides and results in different colored solutions with different λ_max_ values. The method of continuous variation was used to determine the stoichiometry of DQA with ethion, malathion and parathion. In each case, it was found that a 1:1 DQA-OP complex formed. Based on the 1:1 stoichiometry, binding constants were calculated to be 6.5 × 10^4^ M^−1^, 1.1 × 10^4^ M^−1^, and 0.2 × 10^4^ M^−1^ for ethion, malathion, and parathion, respectively.

At the end of the DQA titrations with ethion, malathion and parathion, the solution color had changed from yellow to red-orange, orange and peach-orange, respectively. The same color changes in DQA were also observed when saturation concentrations of OP pesticides were added. No color change was observed when fenthion was added to DQA.

Furthermore, the number and wavelength positions of isosbestic points can be used as a reliable qualitative and quantitative diagnostic tool in the detection and analysis of OPs. For example, Figure 13 shows that parathion and fenthion show no isosbestic points while ethion and malathion each has two. Structurally, parathion and fenthion are similar having three oxygen-bonded groups to thiophosphoryl, while ethion and malathion both have a sulphur group bonded to the thiophosphoryl group.

**Figure 14.**
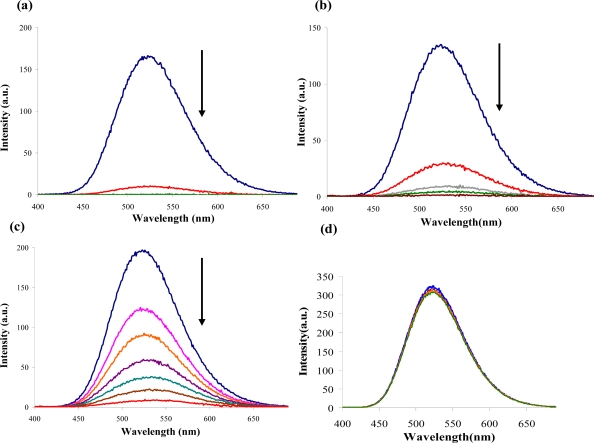
Changes in DQA fluorescence emission spectra upon binding to OPs. (a) titration with ethion, from top to bottom concentration of ethion = 0, 2, 4, 6 μM; (b) titration with malathion, from top to bottom concentration of malathion = 0, 2, 4, 8 μM; (c) titration with parathion, from top to bottom concentration of ethion = 0, 2, 4, 6, 8, 10, 12 μM; and (d) titration with fenthion, with up to 24 μM of fenthion being added with no change observed. The arrow indicates the direction in which the fluorescence intensity change takes place.

The emission spectra of DQA obtained in acetonitrile solvent showed a peak centered at 530 nm. The changes in fluorescence spectra of DQA were measured with the various OP pesticides. Figure 14a shows the results obtained with titration of ethion which showed complete fluorescence quenching of DQA. Similarly, titration of DQA with both malathion and parathion resulted in fluorescence quenching, as shown in Figure 14b and c, respectively. In the case of fenthion, we observed similar behavior to the results obtained with the UV-visible absorbance studies, *i.e.,* titration of DQA with fenthion did not result in significant changes in emission peaks (Figure 14d). We note that in the case of ethion, malathion and parathion, the fluorescence intensity of DQA is quenched but the sensitivity is in the order of ethion > malathion > parathion.

Molecules that provide optical and electrochemical signals are ideal for developing sensors that offer dual signal transductions [60]. Cyclic voltammograms were acquired using a BAS CV50 electrochemical workstation using glassy carbon as the working electrode, a platinum wire as the counter electrode, and Ag/AgCl as the reference electrode. The electrolyte was a 0.1 M solution of tetrabutyl ammonium hexafluorophosphate (TBAPF_6_). DQA was found to have a formal potential (E^0^) at 860 mV *vs*. Ag/AgCl. Changes in the electrochemical waves of DQA with 1 equivalent of the pesticides ethion, malathion, parathion and fenthion were measured. In the case of ethion, malathion and parathion, the DQA-OP complex formed had significantly different redox characteristics relative to DQA. The DQA/ethion complex showed three redox waves at E_1/2_ = −875 mV *vs*. Ag/AgCl, E_1/2_ = −500 mV *vs*. Ag/AgCl and E_1/2_ = +500 mV *vs*. Ag/AgCl. The cyclic voltammogram of the DQA/malathion complex was also different relative to that of DQA; in this case two waves at E_1/2_ = −1,498 mV *vs*. Ag/AgCl and E_1/2_ = −870 mV *vs*. Ag/AgCl) corresponding to DQA-malathion complex were observed.

The formation of a DQA/parathion complex also demonstrated significant changes in the redox behavior (E_1/2_ = −1,072 mV *vs*. Ag/AgCl, E_1/2_ = −773 mV *vs*. Ag/AgCl) in comparison to DQA. As expected, there were no changes in the redox behavior of DQA with the addition of fenthion.

The observed DQA-OP reactions can be explained by Lewis acid-base or nucleophile-electrophile interactions between the quinolinyl nitrogen and the OP phosphorus atoms. Reactions of electrophiles (for example, proton, metal cations, and carbon-based) with 4-dimethylamino styrylazaaromatics occurs exclusively at the ‘ring’ (pyridyl, quinolinyl) nitrogen [61]. This generally results in the formation of the corresponding quaternary pyridinium and quinolinum salts. It is thus reasonable to assume that electrophilic phosphorus reactants will also react preferentially at the azaaromatic ‘ring’ nitrogen. Furthermore, our computational calculations done by GAUSSIAN 03 program suite [62] reveals, as expected, that the electrostatic potential at the quinoline nitrogen is higher relative to the dimethylamino nitrogen.

One common mechanistic pathway for phosphoryl transfer reactions is *via* concerted S_N_2(P) processes in which a nucleophilic attack on phosphorus leads to expulsion of the leaving group. In these S_N_2 scenarios, the reaction rate for the thiophosphoryl transfer is expected to be highly dependent on the leaving group. This in turn will affect the binding constant of the incoming nucleophile. This interpretation is consistent with our results since, for example, it is known that the *p*-nitrophenolate anion of parathion is a much better, more stable leaving group than the phenolate anion of fenthion. Thus, parathion has a stronger binding constant than fenthion to DQA. The interaction of DQA with each OP pesticide relies on the stability of the leaving group - the more stable the OP leaving group, the more likely it will dissociate upon interaction with the nucleophilic DQA quinolinyl nitrogen.

The optical and electrochemical changes of the azastilbene DQA when exposed to ethion, malathion, parathion and fenthion shows the potential of azastilbenes as viable structural motifs for development of multimodal chemosensors. Azastilbenes have demonstrated the capability of distinguishing between various pesticides, which is important for both environmental as well as homeland security applications. Future work on this project to further develop our azastilbene-based multimodal chemosensors for toxic organophosphates and other important toxic analytes is continuing.

## 4. Future Perspectives

Significant progress has been achieved toward the development of fluorescent chemosensors for toxic organophosphorus pesticides and chemical warfare agent mimics. These chemosensors have been demonstrated to be time-effective and more robust that biosensors. It is clear that future improvements in this area will require the design of new fluorescent chemosensors with additional modes for signal transduction. Such sensors will play an important role in minimization or elimination of false-positives. Due to the structural similarity of OP compounds, it is also paramount that the designed sensors must be fabricated such that they are highly selective toward specific OP compounds. Our second generation of azastilbene-based OP sensors will seek to: (*a*) increase sensor multimodality, (*b*) enhance sensor selectivity between oxons and thions, and (*c*) develop robust sensors with real world capability in complex matrices, including aqueous systems.

## Figures and Tables

**Figure 6.**
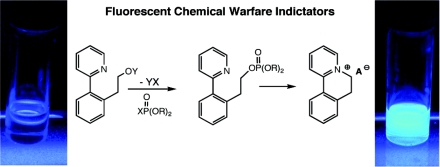
Schematic representation of the chemosensor developed by Zhang and Swager [49]. Reproduced with permission from reference [49], published by American Chemical Society, 2003.

**Figure 15.**
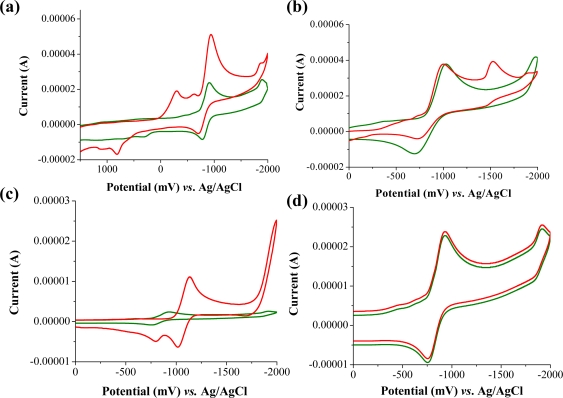
Cyclic voltammograms of DQA before and after addition of (a) ethion, (b) malathion, (c) parathion, and (d) fenthion.

**Table 1.** Common OP pesticides classified by oxon/thion structure and oral LD_50_ toxicities [11–13].

**No**	**OP Name**	**Structure** (Thions: 1–17; Oxons: 18–29)	LD_50_, mg/kg *		**WHO Acute Hazard^§^**	**IARC Carcinogens^‡^**	**U.S. EPA Carcinogens^†^**
			**Oral**	**Dermal**			
**1**	Parathion	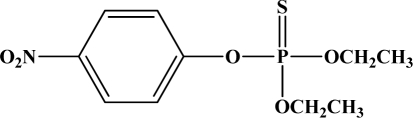	1	21	Ia	3, Unclassifiable	C, Possible
**2**	Fonofos	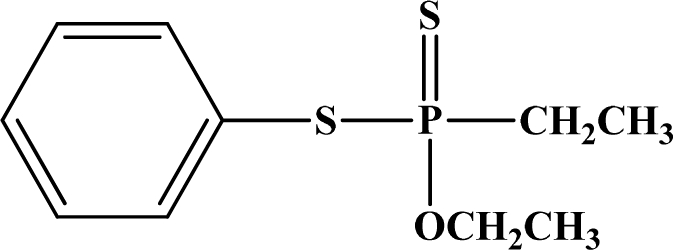	8–17	147	Ia	N/A	E, Unlikely
**3**	Azinphos-methyl	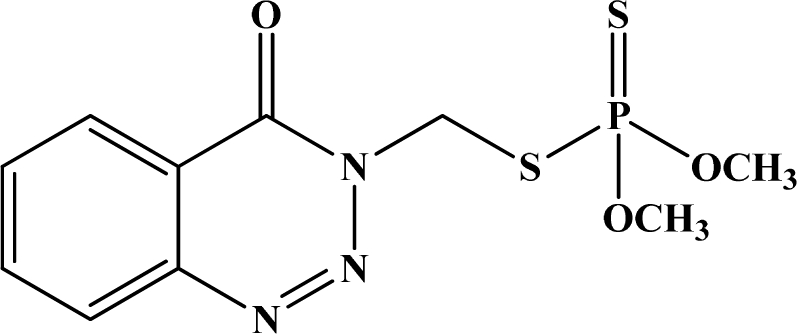	11–13	220	Ib	N/A	Not Likely
**4**	Coumaphos	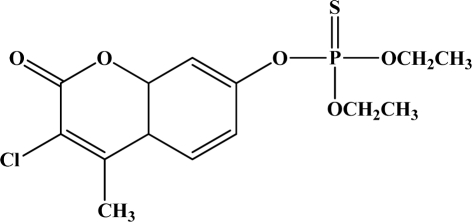	16–41	1,000	Ib	N/A	Not Likely
**5**	Methidathion	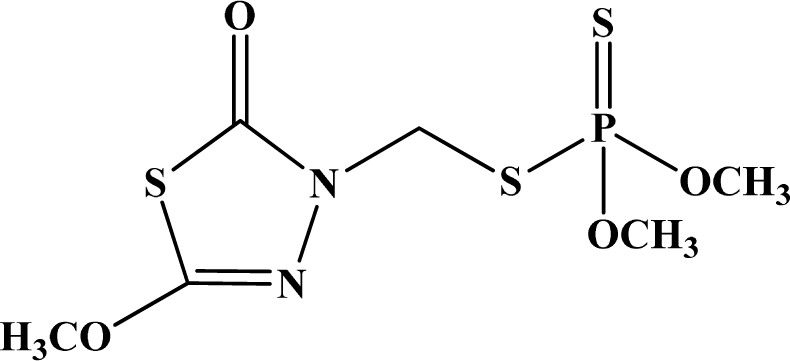	25–48	1,546	Ib	N/A	C, Possible
**6**	Leptophos	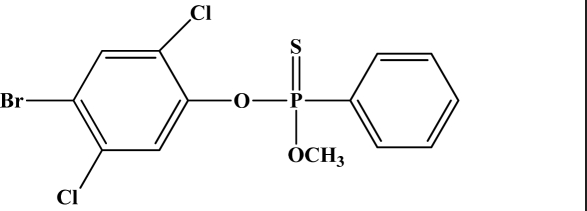	45–53	>800	N/A	N/A	N/A
**7**	Propetamphos	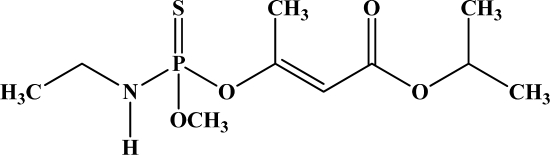	75–82	2,300	Ib	N/A	Not Likely
**8**	Carbophenothion	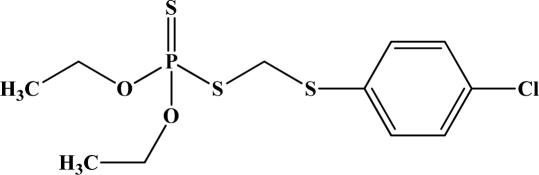	98–120	190–215	N/A	N/A	N/A
**9**	Phosmet	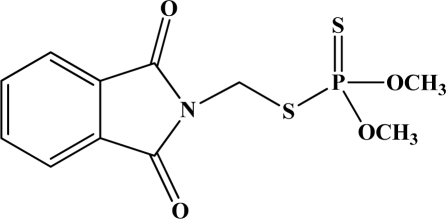	113–160	>1,500	II	N/A	Suggestive
**10**	Chlorpyrifos	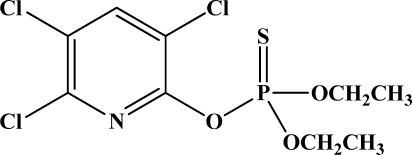	135–165	2,000	II	N/A	E, Unlikely
**11**	Fenthion	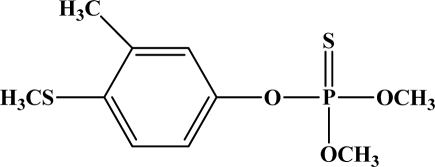	214–245	330	II	N/A	E, unlikely
**12**	Fenitrothion	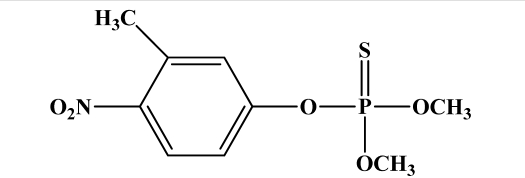	250	>3,000	II	N/A	E, unlikely
**13**	Dichlofenthion	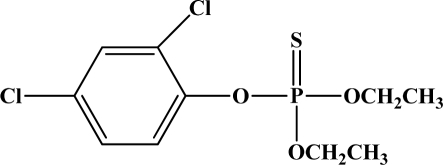	270	6,000	N/A	N/A	N/A
**14**	Dicapthon	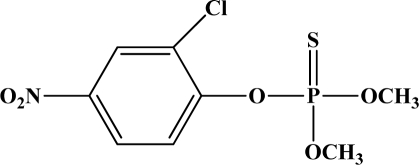	330–400	790–1,250	N/A	N/A	N/A
**15**	Diazinon	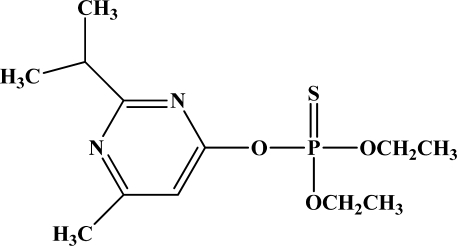	300–850	2,150	II	N/A	Not Likely
**16**	Ronnel	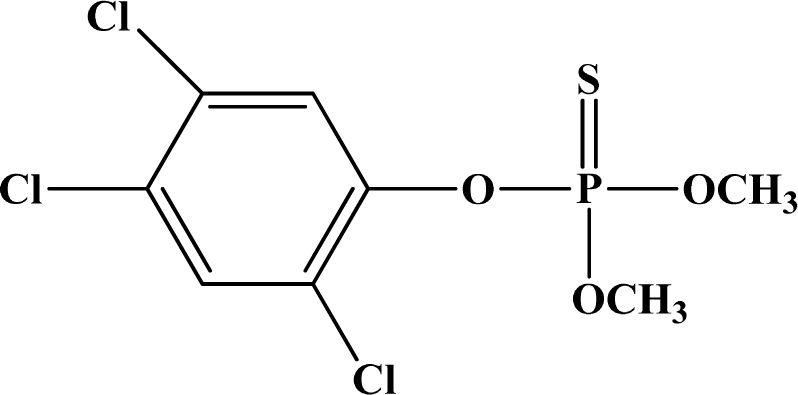	1,250–2,630	2,000	N/A	N/A	N/A
**17**	Malathion	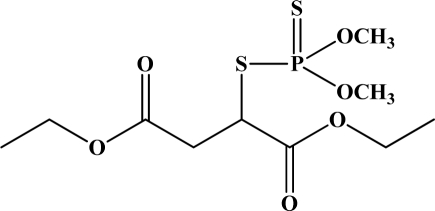	5,400–5,700	>2,000	III	3, Unclassifiable	Suggestive
**18**	Tetraethyl pyrophosphate (TEPP)	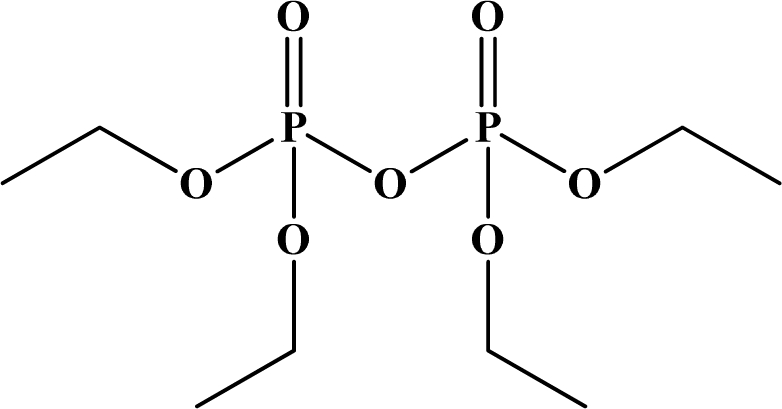	0.5	2.4	N/A	N/A	N/A
**19**	Mevinphos	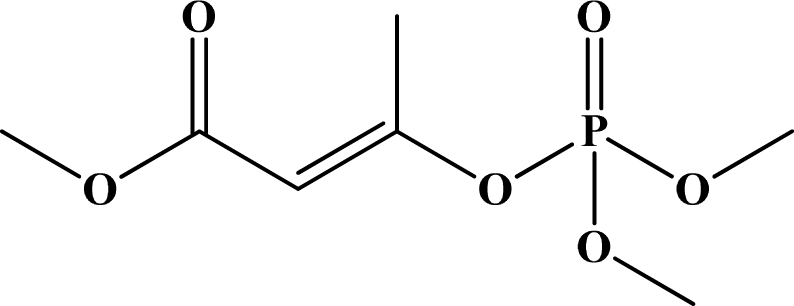	3.7–6.1	4.2–2.7	Ia	N/A	N/A
**20**	Schradan	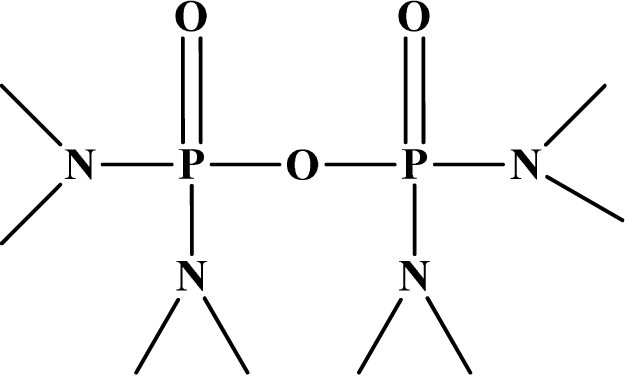	10	15	N/A	N/A	N/A
**21**	Monocrotophos	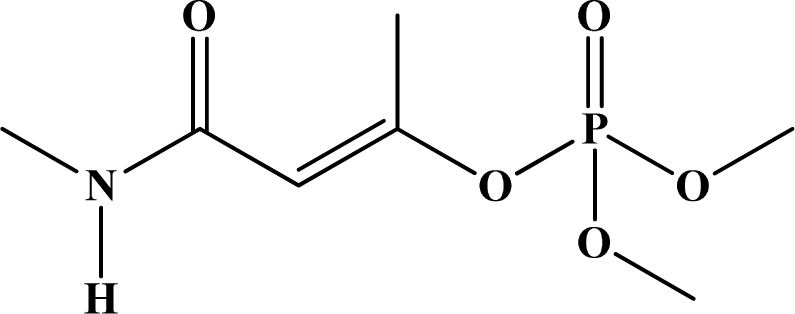	18–20	112–126	Ib	N/A	N/A
**22**	Phosphamidon	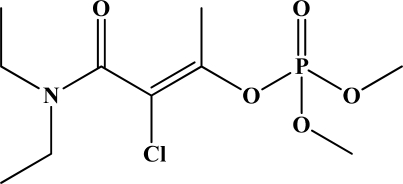	24	107–143	Ia	N/A	C, Possible
**23**	Oxydemeton methyl	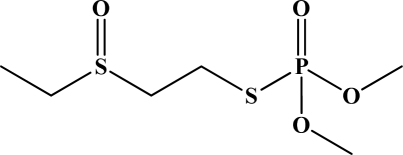	47–52	158–173	Ib	N/A	Not Likely
**24**	Ethoprophos	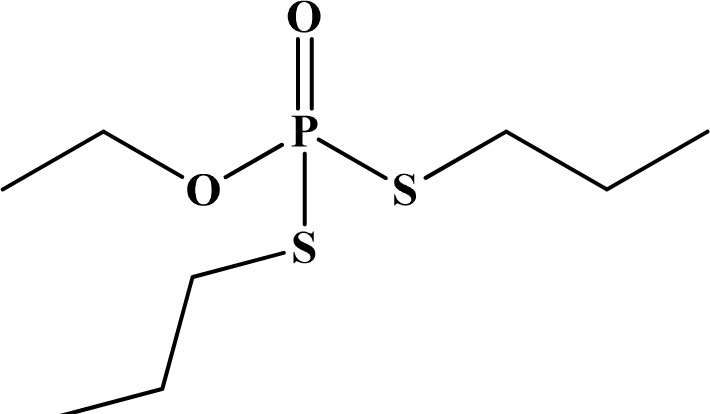	61	26	Ia	N/A	Likely
**25**	Dichlorvos	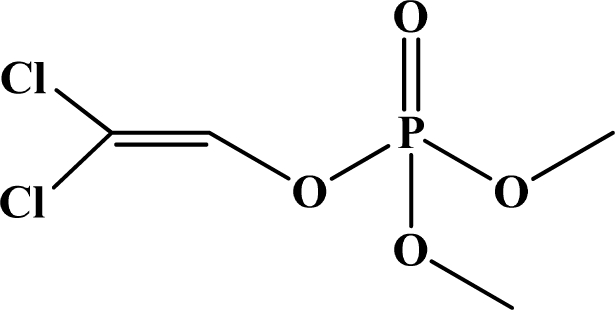	56–80	75–107	Ib	2b, Possible	Suggestive
**26**	Crotoxyphos	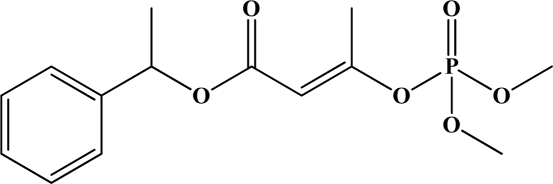	74–110	202–375	N/A	N/A	N/A
**27**	Naled	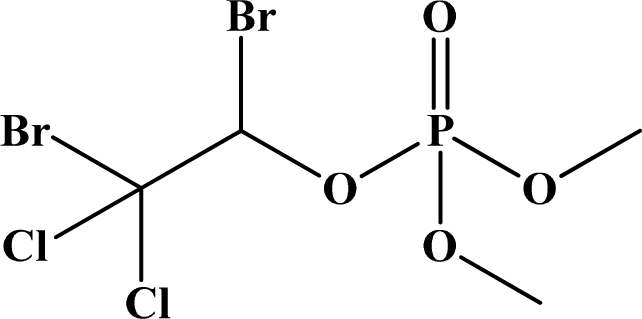	250	800	II	N/A	E, Unlikely
**28**	Tribufos	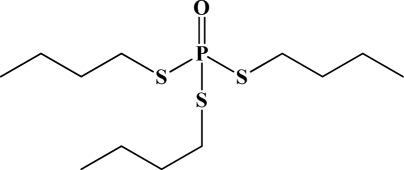	560–630	>2,000	N/A	N/A	Likely (high doses), Not likely (low doses)
**29**	Trichlorfon	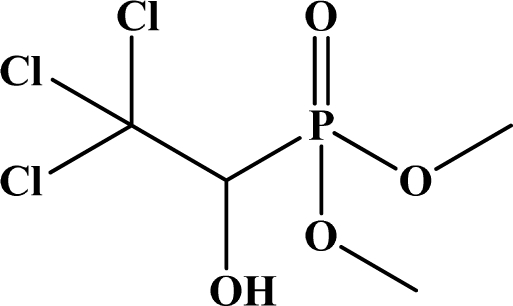	400–800	>2,000	II	3, Unclassifiable	Likely (high doses), Not likely (low doses)

* Toxic interactions of organophosphorus compounds with any given biological system are dose-related. Their toxicity is expressed in terms of the lethal dose (LD) which will kill 50% of the animal species (LD_50_). LD_50_ values are generally expressed as amount per unit.

* Toxic interactions of organophosphorus compounds with any given biological system are dose-related. Their toxicity is expressed in terms of the lethal dose (LD) which will kill 50% of the animal species (LD_50_). LD_50_ values are generally expressed as amount per unit weight (e.g., mg·kg^−1^).

§ WHO = World Health Organization, acute hazard classify: Ia = extremely hazardous to human health; Ib = highly hazardous; II = moderately hazardous; III = slightly hazardous.

‡ IARC = International Agency for Research on Cancer.

† EPA = Environmental Protection Agency.
